# The Completeness of the Operating Room Data

**DOI:** 10.1055/a-2566-7958

**Published:** 2025-06-03

**Authors:** Päivi Nurmela, Minna Mykkänen, Ulla-Mari Kinnunen

**Affiliations:** 1 Department of Health and Social Management, University of Eastern Finland, Kuopio, Pohjois-Savo, Finland; 2 Department of Strategy and Development, Wellbeing Services County of North Savo, Kuopio, Finland; 3 Research Center for Nursing Science and Social and Health Management, Wellbeing Services County of North Savo, Kuopio, Finland

**Keywords:** data, patient data, quality, health information system, operating room

## Abstract

**Background:**

In the operating theater, a large collection of data are collected at each surgical visit. Some of these data are patient information, some is related to resource management, which is linked to hospital finances. Poor quality data lead to poor decisions, impacting patient safety and the continuity of care.

**Objectives:**

The study aimed at evaluating the completeness of the data documented within surgical operations. Based on the results, the goal is to improve data quality and to identify improvement ideas of data management.

**Methods:**

The study was a quantitative evaluation of 33,684 surgical visits, focusing on data omissions. The organization identified 58 operating room data variables related to visits, procedures, resources, and personnel. Data completeness was evaluated for 36 variables, excluding 47 visits that were missing the “Complete” flag. Data preprocessing was done using Python and Pandas, with pseudonymization of personnel names. Data were analyzed using the R programming language. Data omissions were coded as “1” for missing values and “0” for others. Summary variables were created to indicate the number of personnel and procedure and data omissions per visit.

**Results:**

The average completeness of the operating room data was 98%, which is considered excellent. However, seven variables—the start and end date and time of anesthesia, the type of treatment, personnel group, and assistant information—had completeness below the 95% target level. A total of 34% of the surgical visits contained at least one data omission. In the yearly comparison, the completeness values of variables were statistically significantly higher in 2022 compared with 2023.

**Conclusion:**

By ensuring existing quality assurance practices, verifying internal data maintenance and verifying and standardizing documenting practices the organization can achieve net benefits through improved data completeness, thus enhancing patient records, financial information, and management. Improved data quality will also benefit national and international registers.

## Introduction

The management of health care information plays a crucial role in societal decision-making. The quality of the data collected from health information systems is relevant not only for invoicing or information management, 1 but also for patient safety 2 and it has a relation to the image of health care. 3 According to studies, 32% of incident reports that related to information flow and information management were due to missing information. 2

In 2020, 100% of Finland's hospital districts reported full electronic patient record usage in conservative, operative, and psychiatric care. 4 In Finland, the quality and the management of electronic patient records are regulated by law. Several national institutions, such as The Finnish Institute for Health and Welfare (THL) and Finnish Social and Health Data Permit Authority Findata (Findata) monitor, guide, and advice in the implementation of these laws. National standards unify documentation and good quality management practices help public organizations in managing information quality. Organizations must follow these regulations and standards to ensure data accuracy, consistency, and reliability. Regular audits and inspections are conducted to verify that data management practices comply with established standards and regulations. 5

The European Statistics Code of Practice 6 and the Fundamental Principles of Official Statistics by the United Nations Economic Commission for Europe 7 emphasize, among other things, the completeness, accuracy, timeliness, consistency, and comparability of data. In Finland, health data benchmarking is utilized at the national level, against Nordic countries and across Europe, and the Organisation for Economic Co-operation and Development. 5

Data Quality Management involves setting goals, defining responsibilities, and creating and monitoring processes to ensure and improve patient care quality. 1 Finnish health care organizations must report on data quality metrics and to ensure that this information is transparent. 5 Internal quality management ensures the defined quality of all processes and outcomes within the organization. 1 This includes medical, nursing, and administrative guidelines and complies with statutory reporting requirements.

Clinicians need accurate and complete information gathered from patient data. The poor quality of patient information runs the risk of patient safety and continuity of care and does not meet legal obligations or national guidelines. 3 If the patient information that clinicians use is of poor quality, it will lead to poor decisions. 8

The quality of data are more or less well researched in health care. Most of the research is quantitative and focuses on different quality dimensions 9 of which completeness, concordance, and accuracy are dominant. 10 Studies suggest that there are gaps in health care data. Cancer treatment summaries have contained at least one error in 25% of cases and an omission in 22% of cases. 11 Medicine documentation has been missing in 15.1% of the observed cases. 12 Studies concerning operating room find, e.g., that perioperative history was documented in anesthetic records in less than 80% of the sheets 13 and only 2 out of 44 parameters were filled out completely. 14 In Finland, there are few if any studies focusing on operating room data and more robust research is needed to determine the completeness of operating room data.

## Objectives

The purpose of the study was to evaluate the completeness of operating room data in one well-being services county in Finland. Research questions were:

1. How complete is the operating room data?
2. How does the completeness of the operating room data differ between the first 4 months of the years 2022 and 2023?

Based on the results, the goal is to identify ideas for improving data management and, ultimately, enhance data quality. This article represents a part of the Health and Human Services Informatics Master's thesis “The Completeness and the Accuracy of the Operating Room Data,” which was published in the e-repository of the University of Eastern Finland in December 2023 in Finnish. 15

## Methods

The study was a quantitative evaluation study. The data consisted of operating room visits ( *N* = 33,684). Initially, the organization identified a total of 58 operating room data variables from four different datasets. These include visit data variables (1–46), procedure data variables (47–49), resource data variables (50–53), and personnel data variables (54–56). Additionally, there was a variable for used implants (57) and a variable for marking the operation visit as complete (58). Different variables were identifiable and linkable to distinct operating room visit using the visits identifier. The study's focus was on counting data omissions per each operating room visit.

It was presumed that every surgical visit involved personnel and at least one procedure. Certain data fields were omitted from the data quality evaluation because of their specific use cases or lack of validity in the dataset. The study evaluated data quality by focusing on 36 variables: 30 related to visits, 3 related to procedures, and 3 related to personnel ( Table 1 ). In the study data, there were 47 surgical visits with missing “Complete” flags. These visits were excluded from the evaluation of data completeness as all data were missing.

**Table 1:** Data variables of completeness evaluation

Operating room dataset	Variable number	Title	Explanatory note
Visit data	2	SUKUP	Gender
Visit data	4	DG1	Diagnosis 1
Visit data	9	TAKAOS	Sending unit
Visit data	10	TMPYKS ”tpyks”	Operating unit
Visit data	11	LSALI	Operating room
Visit data	12	ANYKS ”ayks”	Anesthesia unit
Visit data	16	HOITOM ”hoitomuo”	Treatment form
Visit data	17	KIIR	Urgency
Visit data	18	TMPTULO ”tpytpvm”	Date of arrival at the operating unit
Visit data	19	TMPKLO ”tpytklo”	Time of arrival at the operating unit
Visit data	20	TMPYAIKA ”tpyaika”	Operating unit time
Visit data	21	SJNRO	Room number in order
Visit data	22	SALIALKU ”salitpvm”	Date of arrival at the operating room
Visit data	23	SALIKLOA ”salitklo”	Time of arrival at the operating room
Visit data	24	SALILOPPU ”salilpvm”	Date of leaving room
Visit data	25	SALIKLOL ”salilklo”	Time of leaving room
Visit data	26	LKESTO ”saliaika”	Operating room time
Visit data	27	ANALKU ”aapvm”	Anesthesia start date
Visit data	28	ANKLOA ”aaklo”	Anesthesia start time
Visit data	29	ANLOPPU ”alpvm”	Anesthesia end date
Visit data	30	ANKLOL ”alklo”	Anesthesia end time
Visit data	31	ANAIKA ”aaika”	Anesthesia time
Visit data	32	TMPALKU ”tpapvm”	Procedure start date
Visit data	33	TMPKLOA ”tpaklo”	Procedure start time
Visit data	34	TMPLOPPU ”tplpvm”	Procedure end date
Visit data	35	TMPKLOL ”tplklo”	Procedure end time
Visit data	36	TMPAIKA ”tpaika”	Procedure time
Visit data	43	AVALMAIKA	Preparation time
Visit data	45	LKTKIIR	Medical urgency
Visit data	46	ERIKOISA	Specialty
Procedure data	47	JNRO	Procedure order number
Procedure data	48	TMPKOODI ”tpkoodi”	Procedure code
Procedure data	49	TMPLAJI ”tplaji”	Type of procedure
Personnel data	54	HNIMI	Personnel name
Personnel data	55	HRYHMA	Personnel group
Personnel data	56	PAVUST	Assistant

The data were delivered to a secure research environment in .xlsx format by a representative of the organization. Preprocessing was primarily done using Python (version 3.9.12) in Visual Studio Code (version 1.75.0, release date 2023-02-01T15:23:45.584Z, electron: 19.1.9, chromium: 102.0.5005.194, node.js:16.14.2, V8: 10.2.154.23-electron.0, OS: Windows_NT x64 10.0.17763, sandboxed: No). Python was used for pseudonymizing personnel names, coding data omissions, and aligning data from separate tabs (visit, procedure, resource, and personnel) to the same visit, as combined dataset, using Pandas. Python and Pandas have been used earlier in research for data quality assessment of patient data. 16 During analysis, personnel data were handled only in pseudonymized form. Data omissions were coded in Python by setting “NULL” or “NaN” values to “1” and other values to “0,” enabling the counting of data omissions.

Surgical visits typically involve multiple procedures, resources, and personnel. Summary variables were created to indicate the number of personnel and procedures per visits identifier and the number of data omissions in different datasets. These summary variables were then aligned with visit data to examine the total number of data omissions per surgical visit. If procedure data were missing, three data omissions were coded for the observation. Similarly, three data omissions were coded for missing personnel data and four for missing resource data. A summary variable “O_MAX” was constructed from the combined data, representing the visit identifier the maximum sum of all possible omissions per visit.

### Analysis

The data were analyzed using the R programming language in the RStudio (version 4.1.3, release date: 2022-03-10, platform: x86_64-w64-mingw32/x64) environment with standard statistical methods. Common metrics such as frequencies, minimum and maximum values, ratios, and central tendencies were used. Variables were coded as Boolean, and their frequencies and modes were analyzed. For counts of personnel, procedures, or resources, the mode was used as the central measure. Summary variables were treated as continuous and analyzed using mean, standard deviation, and standard error. Results were rounded to two decimal places, except for whole numbers, while tables retained nine decimal places for precision. An essential variable was the frequency of data omissions, “P_YHT,” with the average amount of omissions calculated using the maximum amount of omissions variable “O_MAX.” The data omission ratio ranged from 0 (complete data) to 1 (data omission in all columns).

When evaluating the completeness of data, the mean of the data omissions calculated for the variables can vary between 0 and 1, where 0 means data in all rows is present, 1 means data omission in all rows.

This value could be called the incompleteness value of the variable, ICVv, where *xij* *xij* is the binary incompleteness variable i in the j column of the row, ICVvj is the measured value of incompleteness in the j column, and takes values between 0 and 1:



where r is the number of rows and r > 0.

The completeness value, CVv *j* , determines the completeness of data in column j:



In the same principle, the mean of the data omissions calculated for surgical visits can vary between 0 and 1, where 0 means data in all columns, 1 means data omission in all columns. This value could be called the incompleteness value for surgical visit (ICVsv), where O_SUMij is the value of binary calculated data omissions sum “O_SUM” in row I and column j, O_MAXi is the maximum of data omissions in row *i* :



where O_MAXi > 0, while “O_MAX” > 0

Completeness value, CVsvi, determines the completeness of data in row i:



The number of data omissions “O_YHT” was not comparable between surgical visits due to varying numbers of procedures and personnel. Quality evaluation was done by examining the completeness values of observations and variables.

Data omissions and completeness values of variables were compared between 2022 and 2023. The analysis was performed for surgical visits conducted in the first 4 months. 8,943 observations from each year were used for comparison. The distribution of completeness values was examined and compared across the years. The Kolmogorov–Smirnov goodness of fit test is regarded as a key analytical method for electronic health record datasets. 16 It was used to compare the distributions of operating room data completeness between the datasets from 2022 to 2023.

To report the results, descriptive statistical tables were saved as .csv files from the secure environment. These .csv files were then opened in LibreOffice and combined with box plots, histograms and distribution function graphs to illustrate the distribution of observations. The reference level in the figures was the mean or the target completeness level.

#### Criteria

There are no established standards or criteria for the quality of data in health care. This is typical in health informatics research, where the best available approximation of the “truth” must be used. 17 In this study, the criteria for data completeness include operating room data variables that are mandatory.

#### Standards

Defining the completeness of operating room data are challenging. Legally, patient records must be immutable and error-free, containing necessary and sufficient information for treatment. 18 The study assumes that operating room data in surgical visits are complete for mandatory variables.

The standards for evaluation are based on commonly used assumptions in science research. A 95% confidence level is typically regarded as sufficient in statistical research. Therefore, the study concludes that 95% completeness of operating room data are considered adequate. Some studies indicate that clinical research views a data deficiency of over 10% as potentially biased 19 and a deficiency of over 40% as more suitable for generating hypotheses rather than confirming them. 20 The study aims for a generally accepted confidence level for data quality, rating ≥ 95% coverage as sufficient and excellent, 90 to 95% as good, below 90% as fair, and below 60% as poor.

#### Ethics

The study adhered to relevant legislation and good scientific practice as guided by the Finnish National Board on Research Integrity. 21 The research was conducted honestly and with general diligence and accuracy from the research work to the presentation and evaluation of results. A research permit was obtained in accordance with the organization's practices. Data handling and analysis were conducted in a secure environment, adhering to the organization's data protection protocols. Access to the data was restricted to designated individuals.

Data retrieval from the patient information system was performed by a specialist from the organization. The researcher could request clarifications regarding data retrieval and variable listing as the study progressed. Patients were not directly identifiable from the data, as cases were handled using unique visit identifiers. The researcher pseudonymized personnel data before data merging and analysis. After this, the personnel were no longer directly identifiable from the data. Results were transferred from the secure environment to the researcher's computer anonymously, following the environment's practices and Findata's 22 guidelines.

The presentation of results follows ethical research practices while considering the public image of the organization. The research was conducted independently, and the researcher bears all responsibilities and duties related to the study.

## Results

**Fig. 1:**
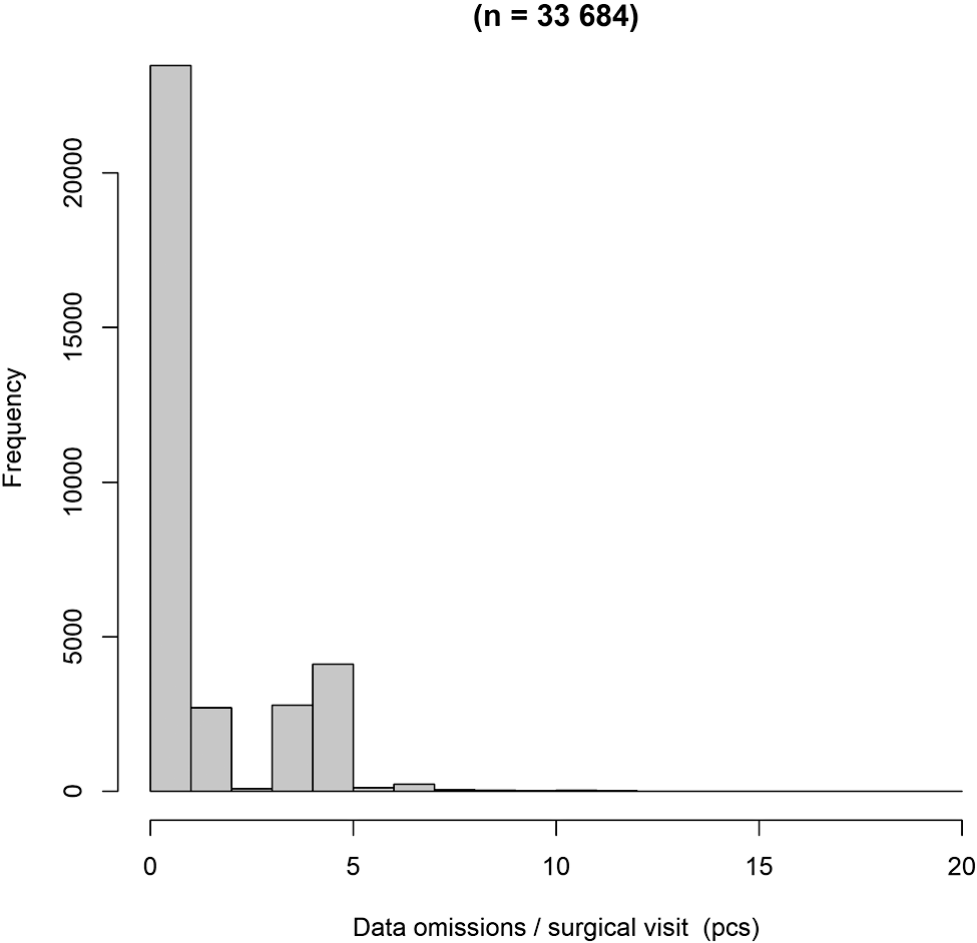
Data omissions per surgical visit.

The data completeness evaluation assessed the completeness of 36 mandatory operating room data variables. The total number of data omissions for the selected variables ranged from 0 to 20. All intervention and resource data were present in 66% (22,251/33,684) of the surgical visits. Typically, there were 0 data omissions per surgical visit, with an average of 1.26 data omissions ( Fig. 1 ). However, the total number of data omissions between observations is not comparable between observations, as they include different numbers of procedures and personnel.

**Fig. 2:**
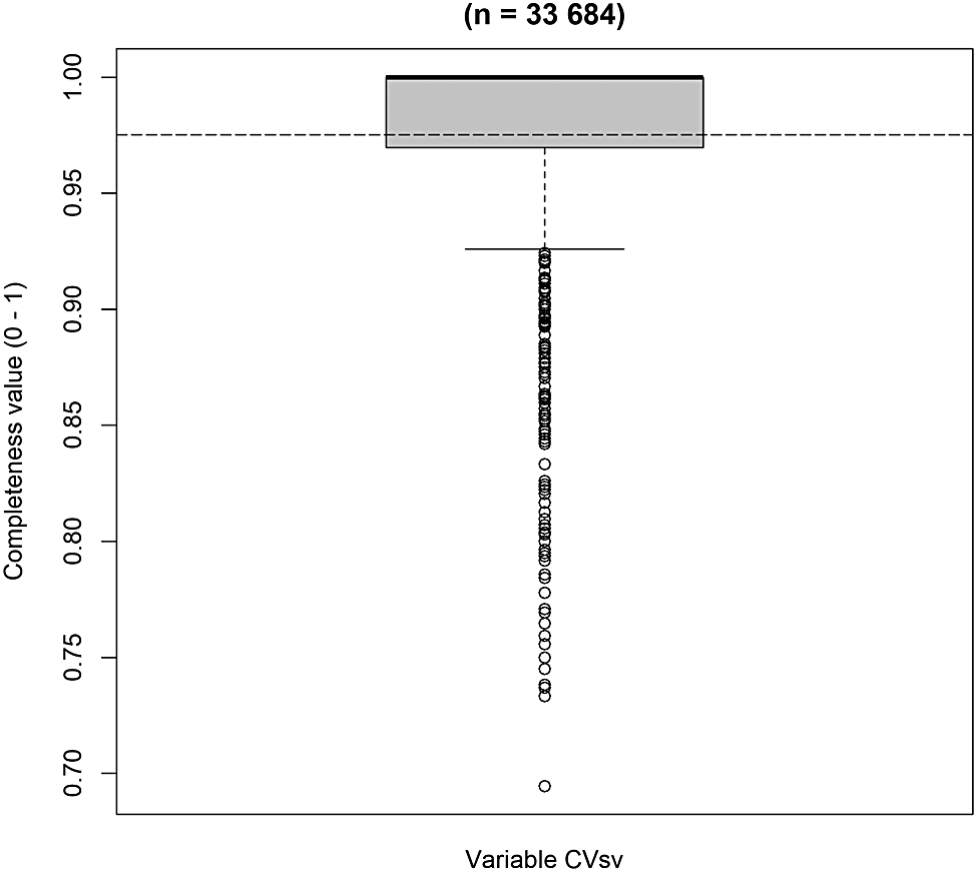
Deviation boxplot of surgical visits completeness value.

The mean surgical visit's completeness value in the combined data was 0.98 (0.975311), ranging from 0.69 to 1.00 ( Fig. 2 ). The distribution of the surgical visit's completeness value is skewed to the left and bimodal (two-peaked), thus not satisfying the normal distribution assumption.

**Fig. 3:**
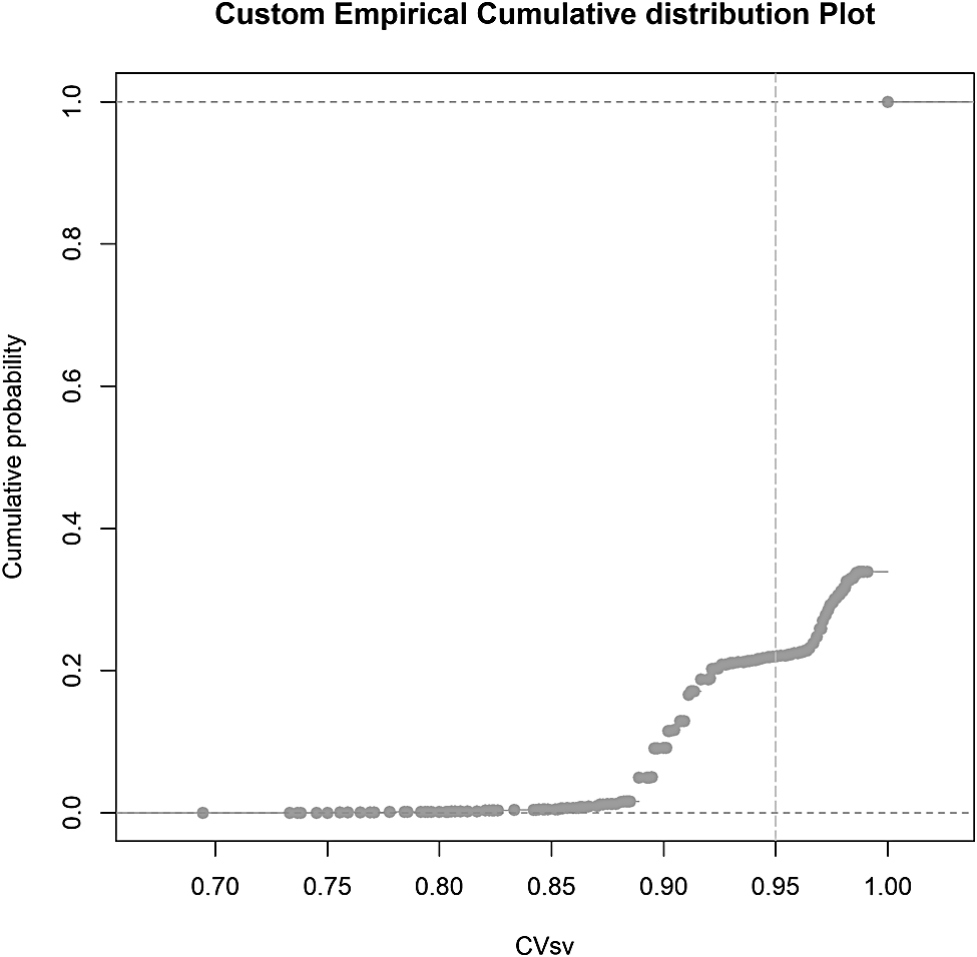
The empirical distribution function of the surgical visit's completeness value.

The empirical distribution function of the surgical visit's completeness value shows that approximately 20% of the data falls below the target level drawn as a reference ( Fig. 3 ). The function tightens as it approaches the maximum of the completeness value, including 34% of the data. The graph of the function then rises without any intermediate observations directly to a single point with a completeness value of 1, containing approximately 65% of the data. At completeness values of 0.88 and 0.97, the sample size function rises more steeply, reflecting a stepwise increase in the coverage of the observed data.

**Fig. 4:**
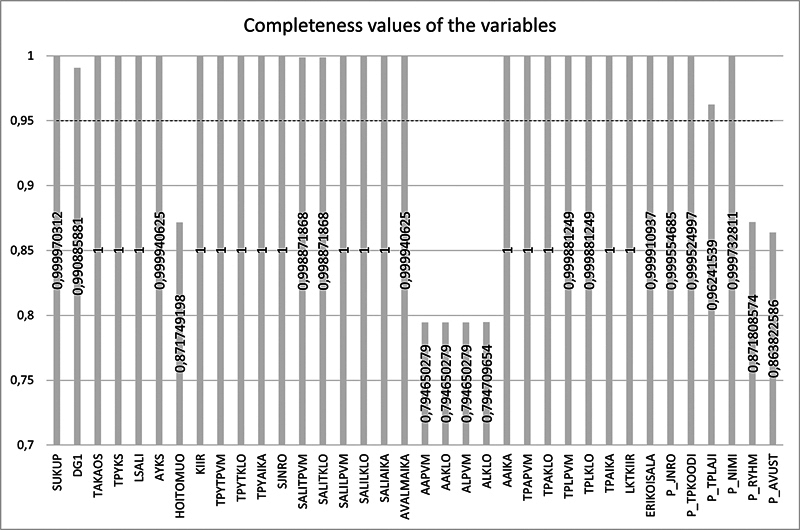
Completeness values per variable.

Of the variables, 16 did not contain a data omission in any surgical visit during the period. The number of data omissions per variable ranged from 0 to 6917. The completeness value of the variables ranged from approximately 0.79 (0.794650) to 1. The completeness value for the seven variables does not reach the desired 95% completeness. Of these, the start and end date and time of anesthesia fell below 80% completeness. Type of procedure, personnel group, and assistant information reached a completeness of more than 85%, but also fell short of the good level of >90% ( Fig. 4 ). In line with the standardization of the survey, the above seven variables were given a failing grade in the coverage assessment.

### Comparison of the Early Years 2022 and 2023

**Fig. 5:**
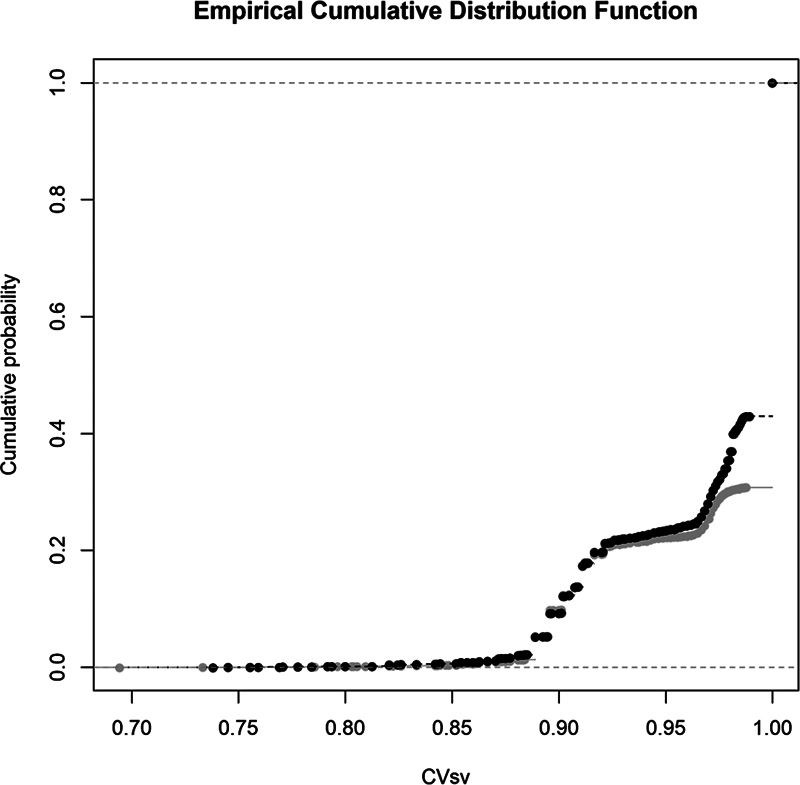
The empirical distribution function of the years 2022 versus 2023 (black = 2022, gray = 2023).

A comparison between the first 4 months data ( *n* = 8 943) in 2022 and 2023 showed that the median number of total data omissions for the samples was different, 60 (2022) and 63 (2023). As this is a total sample, the samples are from a different population, so it is sufficient to compare the medians to show that the samples are different. There was no difference in the median value of the completeness of the surgical visits, but the mean, minimum, skew, and the kurtosis of the distribution were different between 2022 and 2023. Distribution function ( Fig. 5 ) illustrates the difference between the distributions of the samples. To confirm this finding, two sample Kolmogorov–Smirnov tests were done with D^+ = 0.0057028 for a *p* -value of 0.7476. The test showed that the completeness values for the 2022 observations are statistically significantly higher than the completeness values for the corresponding time point in 2023.

The Kolmogorov–Smirnov test assumes a normal distribution of sample means. The sample means of the data were not normally distributed and therefore a nonparametric method was used for testing. According to the two-sample Wilcoxon rank-sum test, the medians of the completeness values of the 2022 and 2023 observations are statistically significantly different (W = 43607738, *p* -value < 2.2e −16).

## Discussion

The aim of this study was to evaluate the completeness of operating room data in one well-being services county in Finland. The study confirms previous research indicating data deficiencies in health care records. Specifically, it found that 34% of operating room data in surgical visits had data omissions, which is higher than the deficiencies found in cancer patient summaries 11 and anesthesia medication records. 12 The study's dataset was significantly larger, with 33,684 observations, compared with the above-mentioned studies. Operating room data had 98% completeness, which is better than in studies with manually filled anesthesia forms. In the study performed in Ethiopia, anesthesia forms had poor completeness, with medication documented in 91% of cases, preoperative information in less than 80%, and follow-up plans in only 70%. 13 In Malaysian study, only two variables were consistently documented in anesthesia forms, with other variables showing varying completeness. 14 This study, however, notes that seven variables did not meet the 95% completeness target level, with the lowest completeness for anesthesia start and end dates and times, indicating possible undocumented anesthesia procedures. Some procedures, like those in cardiology, lack an anesthesia information system, leading to operating room data omissions as the documentation is done elsewhere.

The comparison of data coverage across different years shows that the operating room data of year 2022 ( *n* = 8,943) were documented significantly more completely than of year 2023 ( *n* = 8,943). The organization uses a quality assurance method that automatically sends incomplete surgical visit data to unit supervisors. If there have been changes in quality assurance practices since 2022, it could affect data completeness. It is important to ensure that supervisors continue to receive reports on data deficiencies.

The study identified three critical deficiencies. First, 47 surgical visits lacked “Complete” flags, resulting in no data being transferred to financial management or national registers. Second some surgical visits lacked personnel data, making it impossible to identify the personnel involved in the care. Third some visits lacked procedure data, which is necessary to comply with legal requirements for sufficient information on care provided. Previous studies suggest that the real issue with health data are not its quality but its incorrect placement. Jylhä's research 2 confirms that user errors, such as copying patient information and incomplete entries, are common. Manual documenting poses challenges and is a source of errors. The study concludes that there are gaps in the operating room data and highlights the need for improved data documenting practices.

### Improvement Ideas

The study identifies several areas for improvement to enhance the completeness of operating room data within the organization. First, quality assurance practices for operating room data should be reviewed and updated. That includes reports generated for supervisors on incomplete data and missing entries in “Complete” flags, personnel data, and procedure data. Second, verifying internal data maintenance should be done in a way that all personnel data includes personnel group information and the improvement of documentation practices in the operating room. Third, documenting practices for anesthesia timestamps, “Complete” flags, procedures and personnel should be standardized.

### Strengths and Limitations

The reliability of the study has been considered throughout the study. The purpose, objectives, and research questions have guided the research process. Precise definitions of concepts and variables, along with a comprehensive sampling method, enhance the reliability of this study. The quantitative research approach and examination of data deviations using completeness value is suitable for the quality, size, and previous research on the topic. However, the study's generalizability is limited to the regional level as it only examines the surgical visits of one well-being services county.

The analysis methods were meticulously selected and approved by supervisors. The complete dataset was documented in the original study, and comparisons were made between corresponding periods with equal sample sizes to ensure data comparability.

Data preprocessing was labor-intensive and time-consuming. However, a secure research environment guaranteed a safe and high-quality platform for data analysis. Programming and syntax errors were identified during preprocessing, but the researcher's strong subject knowledge helped in detecting these errors. For instance, issues in visit identifiers caused problems in data merging, which were manually corrected. The research process is thoroughly documented, and the Python code for data preprocessing is available on GitHub, with the R syntax provided upon request, ensuring the study's reproducibility.

As this study primarily focuses on data omissions, it does not provide direct insights into the reasons behind the missing data. It is common practice to categorize missing data into three types: Missing Completely at Random (MCAR), Missing at Random (MAR), and Missing Not at Random (MNAR). 23

The likelihood of missingness in operating room data being classified as MCAR is low. This would imply that the missing data constitutes a random subset of all observations, with no systematic differences between the missing and observed values, which is a theoretical scenario. 23 24 On the contrary, it is more plausible that the missing operating room data falls under the MAR category. It means that the missingness is related to the observed data but not to the unobserved data. 23 24 In this case, there may be systematic differences between the missing and observed values, and the missingness can be fully explained by the observed data values. Additionally, it is possible that some operating room data are MNAR, where the missingness is related to unobserved values given the observed data. 23 This implies that the probability of data being missing depends on the values that are not observed. There may be systematic documentation practices in operating room data that lead to data omissions, such as those associated with certain surgical specialties or types of surgeries.

To determine the types of missingness present in operating room data, further research is recommended. The approach to handling missing data in observational studies is of critical importance; it is advised to employ multiple imputation techniques to complete the data. 25 This will help validate the statistical quality of the study results and ensure unbiased outcomes.

## Conclusion

By ensuring existing quality assurance practices, verifying internal data maintenance and verifying and standardizing documenting practices the organization can achieve net benefits through improved data completeness, enhancing patient records, financial information, and management. Improved data quality will also benefit national and international registers. The study also brings forth opportunities for further research, such as investigating the reasons behind data gaps, examining variables with lower coverage, and comparing operating room data with medical records to evaluate consistency. Additionally, exploring the usability of data in health care and evaluating the perceived net benefits of such data in financial management, administration, and public health institutions would be valuable.
